# Neighbors, Drought, and Nitrogen Application Affect the Root Morphological Plasticity of *Dalbergia odorifera*

**DOI:** 10.3389/fpls.2021.650616

**Published:** 2021-04-08

**Authors:** Li-Shan Xiang, Ling-Feng Miao, Fan Yang

**Affiliations:** ^1^School of Ecological and Environmental Sciences, Hainan University, Haikou, China; ^2^School of Forestry, Hainan University, Haikou, China; ^3^School of Plant Protection, Hainan University, Haikou, China; ^4^Center for Eco-Environmental Restoration Engineering of Hainan Province, Haikou, China; ^5^Key Laboratory of Agro-Forestry Environmental Processes and Ecological Regulation of Hainan Province, Haikou, China

**Keywords:** nitrogen contents, relative competitiveness, root morphological plasticity, root nodules, root system contact model, root system isolated model

## Abstract

In forest systems, neighbor-induced root morphological plasticity (RMP) is species specific and environment dependent. However, related studies on leguminous woody trees remain sparse. The objectives of this study were to evaluate the root morphological response of the leguminous woody *Dalbergia odorifera* T. Chen to different N-fixing niche neighbors under models of root system contact and isolation and to evaluate whether such response can be modified by drought or the application of nitrogen (N). The relationship between root morphology and the relative competitiveness of the whole *D. odorifera* plantlet was also assessed. *D. odorifera* plantlets from the woody Leguminosae family were used as target species and were grown with either identical N-fixing niche *D. odorifera*, the heterogeneous but con-leguminous *Delonix regia*, or the non-leguminous *Swietenia mahagoni*. All plants were grown under two water conditions (100% and 30% field capacity) and two N treatments (no N application and N application). Two planting models (root system contact in Experiment 1, root system isolation in Experiment 2) were applied to neighboring plantlets. The RMP of *D. odorifera* was assessed based on root morphology, root system classification, root nodules, and RMP-related indices. The growth of *D. odorifera* was estimated based on the relative growth ratio, net assimilation rate, and leaf N content. The relative competitiveness of the whole *D. odorifera* plantlet was evaluated through relative yield. The results of Experiment 1 showed that *D. odorifera* had different RMP responses to a different N-fixing niche neighbor with root system contact. The RMP of *D. odorifera* was promoted by a different N-fixing niche neighbor under conditions of drought or N deficiency. Drought improved the RMP of *D. odorifera* exposed to a different N-fixing niche neighbor. N application converted the promoting effect of *D. regia* on RMP to an inhibitory effect under well-watered conditions. Experiment 2 showed that belowground interaction with a different N-fixing niche neighbor may be the only way to influence RMP, as effects of aboveground interaction were negligible. Finally, correlation analysis showed that neighbor-induced RMP might predict the relative competitiveness of the whole *D. odorifera* plantlet under conditions of drought or N deficiency. These findings highlight the influences of neighbors, drought, and N application on the RMP of *D. odorifera* and contribute to understanding neighbor-induced dynamic changes in the root traits of leguminous woody species in forest systems in the context of climate change.

## Introduction

Water and nitrogen (N) are key resources that often determine individual growth, stand productivity, and dynamics of a community structure. Their low availability can induce belowground competition for resources. Roots, as the main organs that obtain soil resources and the first perceptual organs of belowground interaction, are sensitive to the availability of resources and the presence of neighbors and develop a series of adaptive responses in their presence. Root morphological plasticity (RMP) can be used to evaluate the advantages of resources and neighbors in terms of plant relative competitiveness and plant growth; such advantages can be assessed by root biomass distribution, root proliferation, root volume, root density, root length, specific root length (SRL), and mycorrhizal colonization (Lewis and Tanner, 2000; Bennett et al., 2012; Mou et al., 2012; Poorter et al., 2012; Goisser et al., 2016). With varying availability of resources, RMP may be modified and may influence the outcome of competition for resources (Mou et al., 2012; Ma et al., 2014).

Niche complementarity between species would explore a more exhaustive resource compared with niche similarity (Kahmen et al., 2006). Relevant resource-containing niches between belowground competitors determine the root competitive performance of target species (Zuppinger-Dingley et al., 2014). Compared with non-leguminous plants, N-fixing leguminous plants have extensive root systems and can acquire atmosphere-free N by symbiosis with N-fixing rhizobia in root nodules (Andrews and Andrews, 2017). Given the niche complementarity in N-fixing niches between leguminous and non-leguminous plants, leguminous trees have been widely used to facilitate productivity and to improve the tolerance of neighboring non-leguminous trees in mixed plantations (Lithourgidis et al., 2011; Yao et al., 2019). However, empirical evidence of the root plasticity of leguminous woody trees when interacting with different N-fixing niche neighbors is lacking.

Resource competition theory (Tilman, 1981, 1982, 1987), the stress gradient hypothesis (Bertness and Callaway, 1994), and related practices have confirmed that root competitive responses may be modified by the availability of soil resources (McNickle et al., 2014; Goisser et al., 2016; Guo et al., 2016). Previous studies on root responses to competition for resources have focused on either non-leguminous species in a forest ecosystem (Wang et al., 2018a) or leguminous herbaceous crops in an agroforestry ecosystem (Hauggaard-Nielsen et al., 2001; Wang et al., 2017). Leguminous woody species also play a key role in measuring the impact of external conditions on the dynamics of forestry systems (Wang et al., 2018b), especially in the context of climate change. Water and N as main inducers of competition for belowground resources directly affect outcomes of competition. The majority of leguminous species retain homeostasis after the addition of N because of the N absorbed from the soil and that from biological N fixation (Markham and Zekveld, 2007; Guo et al., 2017; Wang et al., 2018b). Conversely, some leguminous species are sensitive to N (Hansen et al., 1992) or water deficits (Sadras et al., 2016). However, the literature on whether the root competitive response of leguminous woody trees varies with drought and N deposition is still lacking, especially for mixed plantations of leguminous trees with different N-fixing niche neighbors.

Plants may detect and identify their neighbors through both aboveground and belowground mechanisms (Chen et al., 2012). The processes of aboveground and belowground competition are interactive and highly interdependent (e.g., on root structure; Bilbrough and Caldwell, 1995). Compared to belowground root competition for nutrients, plants subjected to a combination of aboveground and belowground competition allocate fewer resources to roots (Poorter et al., 2012). Accordingly, RMP is influenced by aboveground interaction (Gundel et al., 2014) and factors such as light and space (Mou et al., 1997). Therefore, we must consider whether the aboveground interaction among neighbors can affect RMP.

To capture more resources, plants can develop RMP to a heterogeneous distribution of nutrients that results from spatio-temporal heterogeneity in a natural soil environment or from resource depletion by neighbors (Mou et al., 1997, 2012; Wang et al., 2018a). The neighbor-induced RMP of a plant is generally estimated from some root-related indices, namely, the root response ratio (RRS) and root relative competition index (RRI; Li et al., 2013, 2018). In addition, foraging strategies based on root traits can be used to determine whole competitiveness (Semchenko et al., 2018). For example, in a mixture of two maize (*Zea mays* L.) genotypes (XY335 and HMY), the higher RRS (RRS > 0) of XY335 reflected a stronger RMP, which ultimately conferred a stronger competitive advantage on XY335 in a mixture of XY335 and HMY (Li et al., 2018). The majority of related studies also emphasize that plants with greater root proliferation can absorb nutrients more quickly under conditions of heterogeneous nutrients. Thus, plants with a higher RMP have a greater whole competitive advantage in a mixture (Semchenko et al., 2018). Traits may be required to predict plant performance and plant–plant interaction (Schroeder-Georgi et al., 2016). Therefore, RMP based on both the RRS and RRI may provide insights into the potential competitiveness of woody legumes with a different N-fixing niche neighbor.

Non-leguminous species in a forest ecosystem develop diverse RMP in response to the varying availability of resources and different resource-containing niche neighbors, especially in the context of climate change. However, most studies on leguminous species have focused on leguminous herbaceous crops in an agroforestry ecosystem. There is little knowledge of leguminous woody trees in a forestry system. N-fixing leguminous woody species have extensive root systems and play an essential role in measuring the impact of external conditions on the dynamics of forestry systems (Andrews and Andrews, 2017; Wang et al., 2018b). Thus, it is critical to understand the RMP of leguminous woody species when interacting with different N-fixing niche neighbors and test whether such RMP varies with drought and N deposition. This would help researchers evaluate neighbor-induced dynamic changes in the root traits of leguminous woody species in forest systems with the increase in extreme climatic events. *Dalbergia odorifera* is a precious leguminous woody tree with rhizobia in its root nodules. It has economic and medicinal value and is endemic to Hainan Island. *Delonix regia* and *Swietenia mahagoni* are tropical woody species with both economic and medicinal value; they are heterogeneous and belong to the same leguminous family as and a different family from *D. odorifera*, respectively. Thus, these three species belong to different N-fixing niches. The objectives of this study were to (1) characterize the effects of different N-fixing niche neighbors on the root morphology of leguminous woody *D. odorifera* with root system contact, (2) test whether the root morphology of *D. odorifera* is influenced by aboveground interaction with different neighbors with root system isolation, (3) determine whether this is influenced by drought and N application, and (4) evaluate whether the relative competitiveness of whole leguminous woody *D. odorifera* with different N-fixing niche neighbors can be predicted by RMP under a given environmental condition.

## Materials and Methods

One-year-old plantlets with approximately the same basal stem diameter and height were selected from a local nursery garden. No significant statistical differences between plantlets were observed. The experimental design was completely randomized, with three factors (water regime, N fertilization, and species competition). Two water conditions [well-watered: 95–100% field capacity (FC), drought: 25–30% FC] and two N treatments (N fertilization and no N fertilization) were set up after 4 weeks of growth. There were 24 treatments with five replicates per treatment in Experiment 1 (Exp1) and Experiment 2 (Exp2). In the well-watered and drought treatments, containers were weighed daily and rewatered to 95–100% FC and 25–30% FC, respectively, by replacing the amount of transpired water. NH_4_NO_3_ as N fertilizer was dissolved in water and then added to containers once a week at 0.4 g each time from April 2018 to July 2018. The experiment lasted 120 days.

### Experiment 1: Interaction With Root System Contact

To assess the RMP of leguminous woody *D. odorifera* with different N-fixing niche neighbors under conditions of drought and N application with root system interaction, we performed a controlled greenhouse experiment. *D. odorifera* plantlets as leguminous woody target species were grown with *D. odorifera* of the same N-fixing niche (intraspecific competition [Do-Do]); *D. regia* that belonging to a heterogeneous but con-leguminous family (*D. odorifera* + *D. regia* [Do-Dr]); and a species from a different family (different N-fixing niche), *S. mahagoni* (*D. odorifera* + *S. mahagoni* [Do-Sm]). One-year-old plantlets were planted in plastic containers (50 × 21 × 16 cm, length × width × height) filled with 15 kg red soil and sand (1:2, v/v). The two individuals in each container were spaced approximately 25 cm apart, and belowground root contact was maintained.

### Experiment 2: Interaction With Root System Isolation

Another experiment was simultaneously conducted to test the effects of aboveground interaction with different neighbors on *D. odorifera* root performance. Unlike in Experiment 1, we divided each container into two sections by placing a plastic partition in the middle of the container. The two plantlets were planted on either side of the plastic partition, and only aboveground interaction was allowed between them. The three planting models related to the N-fixing niche under these conditions of root isolation were one same N-fixing niche (*D. odorifera* + *D. odorifera* [Do/Do]) and two different N-fixing niches (*D. odorifera* + *D. regia* [Do/Dr] and *D. odorifera* + *S. mahagoni* [Do/Sm]). The water and N treatments were the same as those in Exp 1.

### Harvesting and Analysis of Plant Growth

Five *D. odorifera* plantlets from each treatment were randomly selected for the measurement of plant leaf area with a portable area meter (Li-3000C, Li-Cor Inc., Lincoln, NE, United States) and then harvested after 120 days. All harvested *D. odorifera* individuals were separated into leaves, stems, and roots. The leaves and stems were oven-dried at 72°C for 72 h and then weighed. N concentration in the leaves was determined by the semi-micro Kjeldahl method (Mitchell, 1998).

The relative growth ratio (RGR; g g ^−1^ DM day ^−1^) and net assimilation rate (NAR) were used to evaluate the effects of neighbors on *D. odorifera* growth and understand the effects of the aboveground interaction of *D. odorifera* on root plasticity. RGR and NAR were calculated according to formulas described by Radford (1967) as follows:

RGR=lnDM1−lnDM2t2−t1

$$\mathrm{NAR}=\frac{\left({\mathrm{DM}}_{2}-{\mathrm{DM}}_{1}\right)\left(\ln{\mathrm{lA}}_{2}-\ln{\mathrm{lA}}_{1}\right)}{\left(t_{2}-t_{1}\right)\left({\mathrm{LA}}_{2}-{\mathrm{LA}}_{1}\right)}$$,

where DM_1_ and DM_2_ are the total dry mass of *D. odorifera* at t_1_ and t_2_, respectively; LA_1_ and LA_2_ represent the leaf area at t_1_ and t_2_, respectively; t_1_ (days) and t_2_ are the beginning and end of the experiment, respectively. The t_2_–t_1_ represents the duration of the experiment in days.

Relative yield (RY) was expected to account for the relative competitiveness of *D. odorifera* in the different planting models under a given environment. RY was calculated according to a formula described by De Wit (1960) as follows:

$$\mathrm{RY}=Y_{\mathrm{ab}}/Y_{\mathrm{aa}}$$

Here Y_aa_ and Y_ab_ the are average biomass or nutrient content of the harvested organs of *D. odorifera* in the monoculture model (with the same N-fixing niche neighbor) and mixture model (with a different N-fixing niche neighbor), respectively. When RY > 1.0, *D. odorifera* is more stimulated by a different N-fixing niche neighbor than by the same one. That is, different N-fixing niche neighbors confer competitive advantages on *D. odorifera*. In addition, a greater value indicates a stronger effect on *D. odorifera* biomass from a different N-fixing niche neighbor.

### Determination of Root Traits

Harvested *D. odorifera* roots were washed with running tap water and then carefully separated from neighboring roots. The total biomass of the intact root was measured and then scanned with a root scanner (Epson Perfection 1600 Pro, Model V700, Epson, Tokyo, Japan). Afterward, root length, root surface area, root diameter, root volume, root tips, branching number, and root classification (coarse roots, diameter ≥ 2 mm; fine roots, diameter < 2 mm) were analyzed with WinRHIZO (Regent Instruments, Quebec, Canada). Fresh nodules were carefully removed from *D. odorifera* roots and classified according to size as large (diameter ≥ 2 mm) or small (diameter < 2 mm). These nodules were then counted and weighed. Meanwhile, the ratio of nodule biomass to total root biomass (fresh weight) was calculated. The *D. odorifera* root samples were then collected, dried to a constant weight at 70°C, and weighted. In addition, SRL (the ratio of total root length to root dry weight), specific branching density (SBD; the ratio of root branching number to root dry weight), and specific root tip number (SRN; the ratio of root tip number to root dry weight) was calculated.

The RRI, which can be used as an indicator of neighbor-induced root investment, was calculated by a metric modified from Jolliffe (2000) as follows:

$$\mathrm{RRI}=\left({\mathrm{RB}}_{\mathrm{ab}}-{\mathrm{RB}}_{\mathrm{aa}}\right)/{\mathrm{RB}}_{\mathrm{aa}},$$

where RB_aa_ and RB_ab_ represent the root dry biomass of *D. odorifera* interacting with the same N-fixing niche neighbor or a different N-fixing niche neighbor, respectively, under a given environment. If the root dry biomass of *D. odorifera* interacting with the same N-fixing niche is the same as that of *D. odorifera* interacting with a different N-fixing niche neighbor, then RRI = 0. If RRI > 0, then *D. odorifera* benefits more from a different N-fixing niche neighbor than from the same N-fixing niche neighbor and invests more biomass to the root. If RRI < 0, the opposite is true. A higher RRI indicates greater promotion of root investment of *D. odorifera* from a different N-fixing niche neighbor.

The RRS was expected to explain the extent of root proliferation (coarse, fine, and total roots) to various neighbors under given water and N application conditions. RRS was calculated according to a formula modified from Li et al. (2013) as follows:

$${\mathrm{RRS}}_{a}=\Sigma \;\left[\left({\mathrm{RL}}_{\mathrm{ab}}-{\mathrm{RL}}_{\mathrm{aa}}\right)/\left({\mathrm{RL}}_{\mathrm{ab}}+{\mathrm{RL}}_{\mathrm{aa}}\right)\right]/n,$$

where RRS_a_ represents the RRS of *D. odorifera* to different neighbors; RL_aa_ and RL_ab_ are the root lengths of *D. odorifera* with conspecific competition (aa) and heterospecific competition (ab) under the given water and N application conditions, respectively; and n is the number of (RL_ab_ – RL_aa_)/(RL_ab_ + RL_aa_) values. In this study, n equals 5, because five replicates (five random individuals) were set in each species competition treatment. The root proliferation of *D. odorifera* was expected to be promoted by competition with a heterogeneous neighbor when RRS_a_ > 0 and diametrically suppressed when RRS_a_ < 0. A greater RRS_a_ represents a stronger effect on the root proliferation of *D. odorifera* from a heterogeneous interaction.

### Statistical Analysis

SPSS 19.0 was used for the statistical analysis. All data were tested for a normal distribution and homogeneity of variance before analyses. Multivariate analysis of variance was used to evaluate the effects of the interaction of water, N application, and competition. To estimate the correlations between the RY of the whole *D. odorifera* plantlet and RRI, RY of the roots, and RRS of total roots, we performed a correlation analysis. Differences were considered significant at *p* < 0.05.

## Results

### Effects on Root Morphological Traits

**Figure 1:**
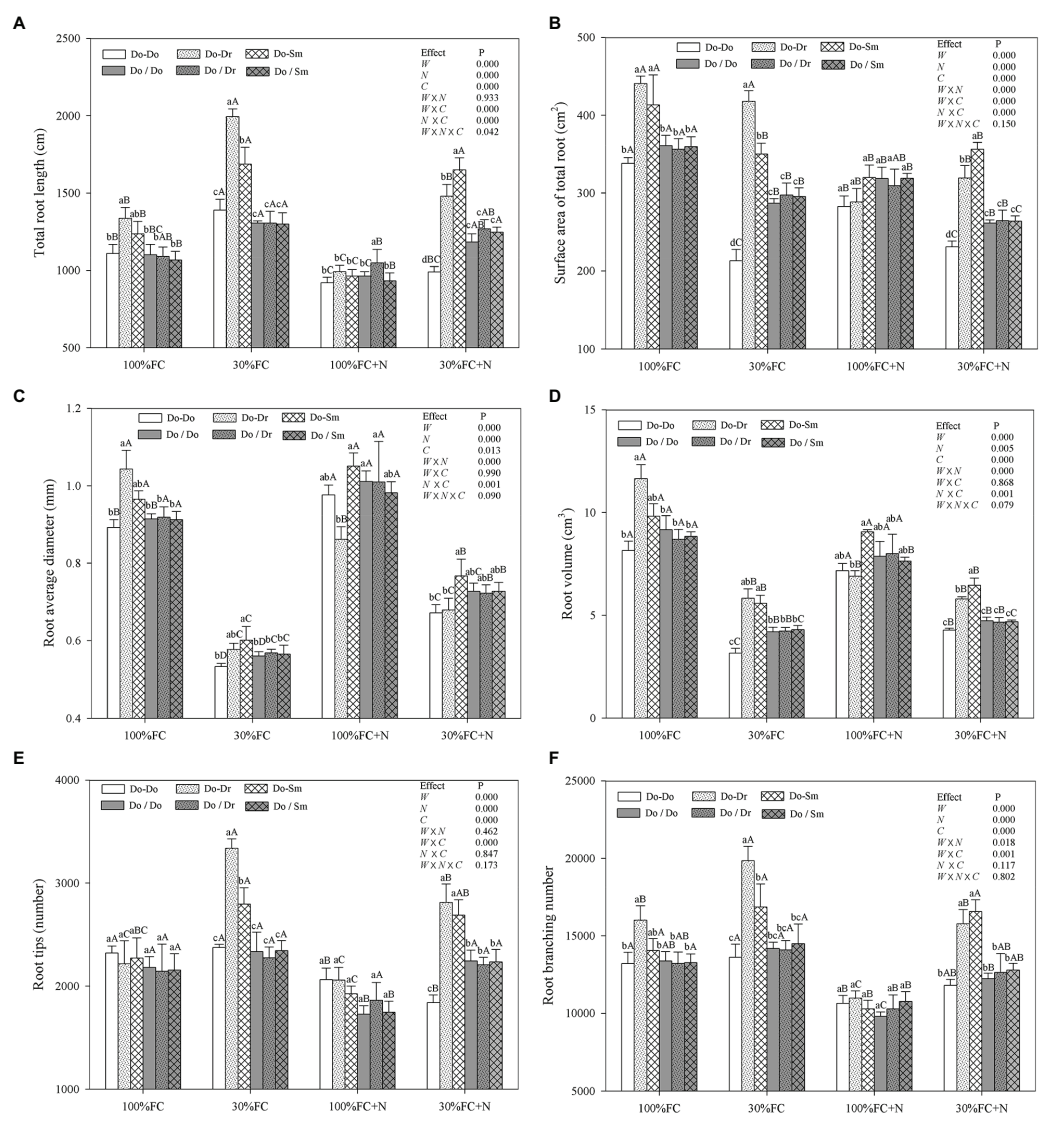
Effects of water, N fertilization levels, and species competition models on total root length **(A)**, surface area of total root **(B)**, root diameter **(C)**, root volume **(D)**, root tips **(E)**, and root branching number **(F)** of *D. odorifera*. Means ± SEs, *n* = 5. Different lowercases in each graph indicate significant difference among different competitive models (competition between *D. odorifera* and various N-fixing niche neighbors) under the same water and N application according to Duncan’s tests (*p* < 0.05). Different uppercases in each graph indicate significant difference among different water and N fertilization application in the same competitive models according to Duncan’s tests. 100% FC, 100% field capacity; 30% FC, 30% field capacity; 100% FC+N, 100% field capacity and N fertilization treatment; 30% FC+N, 30% field capacity and N fertilization. Do-Do, Do-Dr, and Do-Sm indicate *D. odorifera* planted with *D. odorifera*, *D. regia,* and *S. mahagoni* in the root system contact models, respectively; Do/Do, Do/Dr, and Do/Sm indicate *D. odorifera* planted with *D. odorifera*, *D. regia* and *S. mahagoni* in the root system isolation models, respectively. W, water factor effect; N, nitrogen factor effect; C, species competition models factor effect; W × N, interaction effect of water and nitrogen factors; W × C, interaction effect of water and species competition models factors; N × C, interaction effect of nitrogen and species competition models factors; W × N × C, interaction effect of water, nitrogen, and species competition models factors. Multivariate analysis of variance (Multi-ANOVA) was conducted to evaluate the influence of different factors and their interaction effects.

In the root system contact model, the root length, root surface area, root diameter, root volume, and root branching number of *D. odorifera* plantlets exposed to interspecific competition (Do-Dr and Do-Sm) were higher than those of plantlets exposed to intraspecific competition (Do-Do) under both the 100% FC and 30% FC conditions (Figure 1). Drought-stressed *D. odorifera* plantlets showed significantly higher root length, root branching number, and root tips but smaller root diameter and root volume compared to the well-watered plantlets (Figures 1A,C–F). In addition, drought-stressed *D. odorifera* in the Do-Dr and Do-Sm models showed a greater increase in root length, root branching number, and root tips and a smaller decrease in root diameter and root volume compared to plantlets in the Do-Do model. N application decreased the root length, root surface area, root tips, and root branching number under the 100% FC condition (Figures 1A,B,E,F). Moreover, *D. odorifera* in the Do-Dr model showed the greatest decrease in these parameters. *D. odorifera* in the Do-Sm model showed the largest root diameter and root volume. The smallest values among the competition models were found in the Do-Dr model under the 100% FC+N condition with root system contact. N application increased the root diameter and root volume of *D. odorifera* in all competition models but decreased the root length, root surface area, root tips, and root branching number of plantlets under the 30% FC condition. In addition, a greater decrease in the root length, root surface area, root tips, and root branching number of *D. odorifera* was observed in the Do-Dr model compared to the Do-Do and Do-Sm models. *D. odorifera* in the Do-Sm and Do-Do models had the largest and smallest root length, root surface area, root diameter, root volume, root tips, and root branching number among all competition models under the 30% FC+N condition (Figure 1). Root morphological traits were significantly affected by water × N, water × competition, and N × competition interactions. The water × N × competition interaction significantly affected root length.

In the root system isolation model, few differences in root length, root surface area, root diameter, root volume, root tips, or root branching number among the competition models were found among the treatments (100% FC, 30% FC, 100% FC+N, 30% FC+N; Figure 1). Under the 100% FC and 30% FC conditions, the root length, root surface area, root diameter, root volume, and root branching number of *D. odorifera* plantlets with root system isolation were significantly lower than those of plantlets with root system contact in interspecific competition (Do/Dr vs. Do-Dr, Do/Sm vs. Do-Sm; Figures 1A–D,F). However, these parameters (except the insignificant difference in root length) were higher in *D. odorifera* plantlets with root system isolation than root system contact in the intraspecific competition (Do/Do vs. Do-Do). Under the 100% FC+N condition, *D. odorifera* in the Do/Dr and Do/Sm models had higher and lower root diameter and root volume in the root system isolation model than the root system contact model, respectively (Do-Dr, Do-Sm). Under the 30% FC+N condition, *D. odorifera* in the Do/Do model showed a higher root length, root surface area, root volume, root tips, and root branching number than in the Do-Do model, but lower values for these parameters than plants in interspecific competition (Do/Dr vs. Do-Dr, Do/Sm vs. Do-Sm) were found in the root system isolation model than in the root system contact model (Figures 1A,B,D–F).

**Figure 2:**
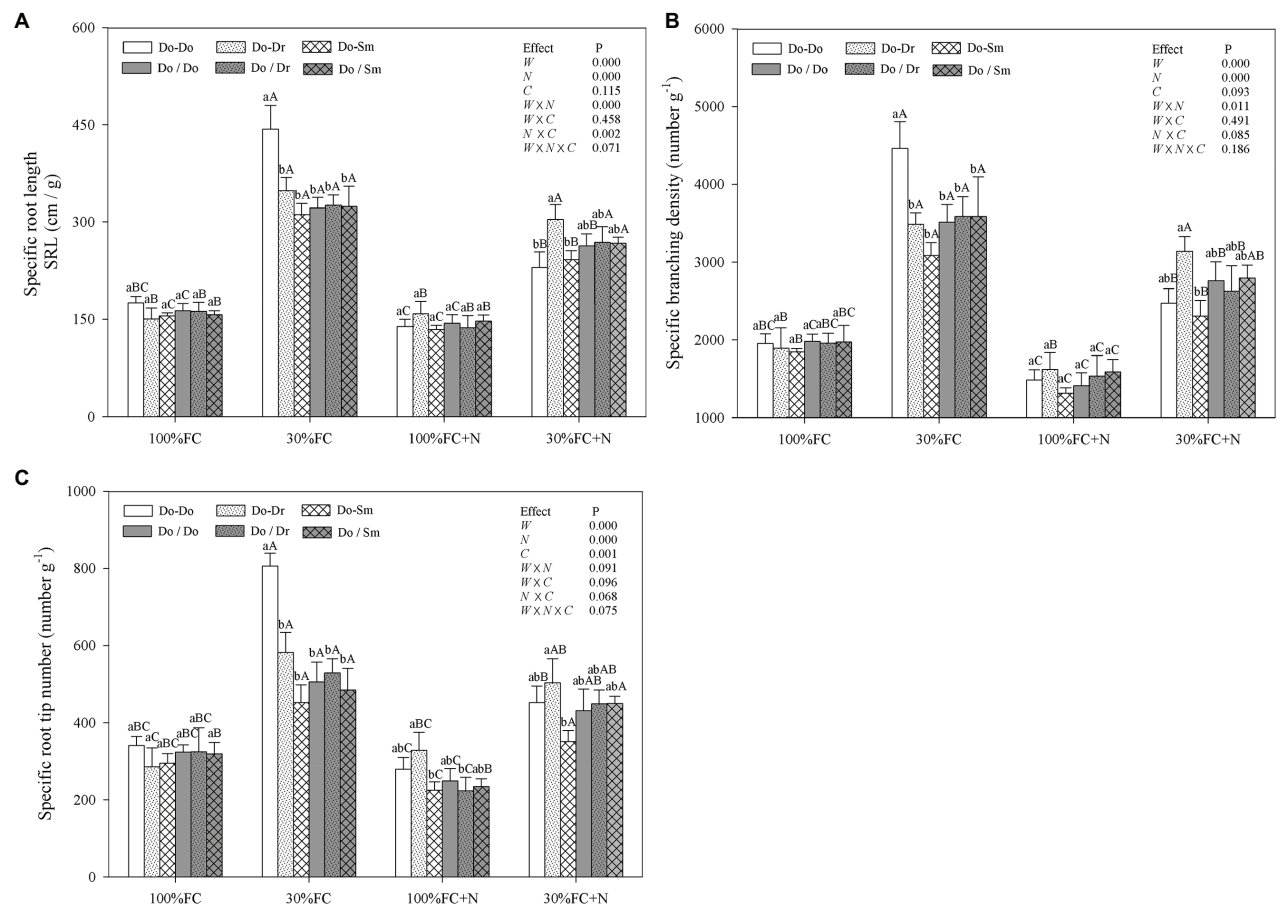
Effects of water, N fertilization levels, and species competition models on specific root length **(A)**, specific branching density **(B)**, and specific root tip number **(C)** of *D. odorifera*. Different lowercases in each graph indicate significant difference among different competitive models (competition between *D. odorifera* and various N-fixing niche neighbors) under the same water and N application according to Duncan’s tests (p < 0.05). Different uppercases in each graph indicate significant difference among different water and N fertilization application in the same competitive models according to Duncan’s tests. For abbreviations explanation of treatments (species competition models, water, and N fertilization), data description, and statistics are the same as shown in Figure 1.

In the root system contact model, drought stress increased the SRL, SBD, and SRN of *D. odorifera* in all competition models (Figure 2). These parameters showed a greater increase in the Do-Do model than in the Do-Dr and Do-Sm models. Moreover, drought-stressed *D. odorifera* in the Do-Do model exhibited the highest SRL, SBD, and SRN among all competition models. N application under well-watered conditions (100% FC) decreased the SBD and SRN of *D. odorifera* in all competition models, except the SRN in the Do-Dr model. Furthermore, *D. odorifera* in the Do-Sm model showed the lowest SRN among all competition models under the 100% FC+N condition. N application under drought stress decreased the SRL, SBD, and SRN of *D. odorifera* in the Do-Do and Do-Sm models; rare variations were found in the Do-Dr model. Moreover, *D. odorifera* in the Do-Sm model exhibited the lowest SBD and SRN among all competition models (Figures 2B,C). However, insignificant differences in the SRL, SBD, and SRN of *D. odorifera* among all competition models were observed in all treatments in the root system isolation model (Figure 2). Finally, water × N and N × competition interactions significantly affected the SRL.

### Effects on Root System Classification

**Figure 3:**
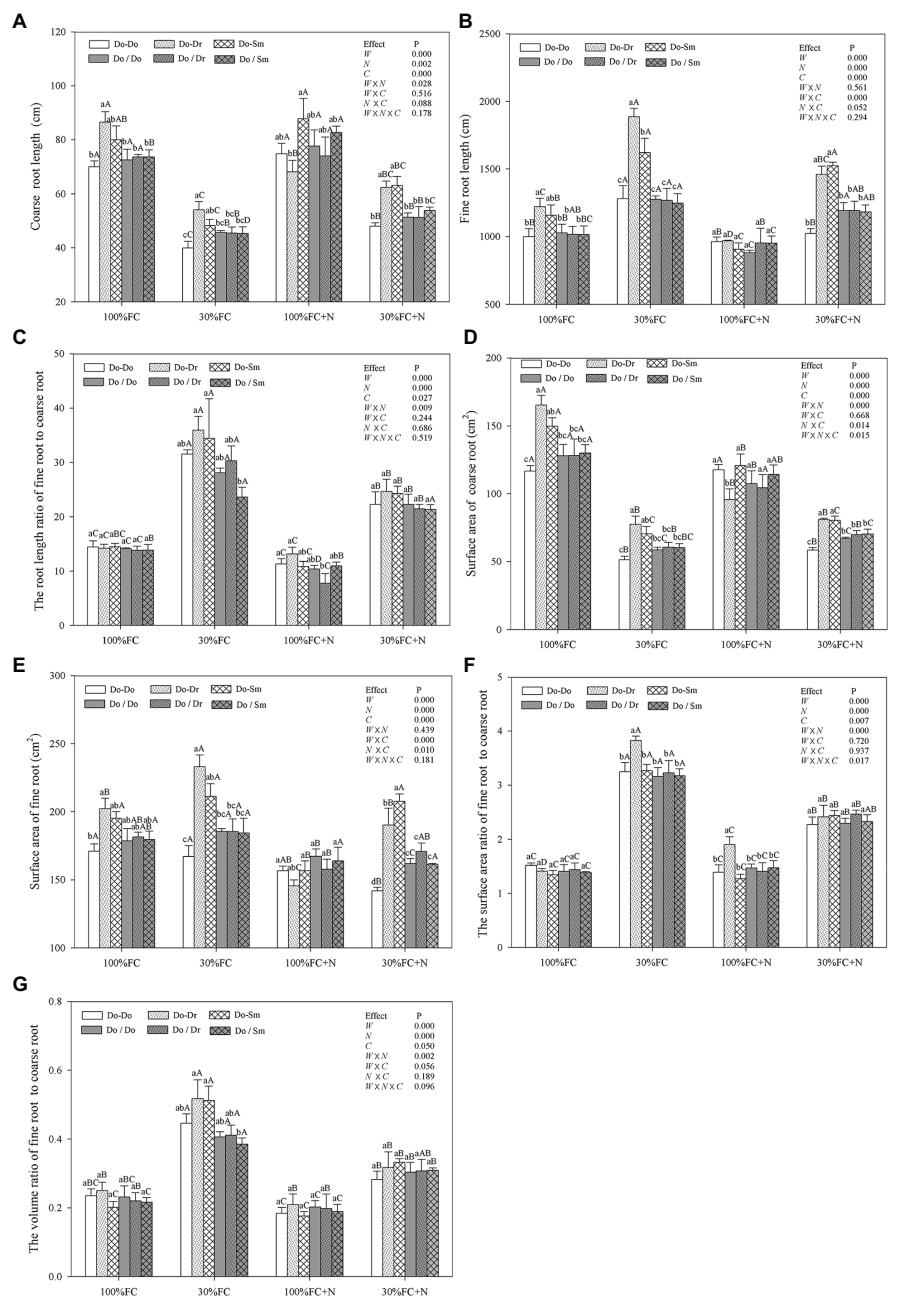
Effects of water, N fertilization levels, and species competition models on coarse root length **(A)**, fine root length **(B)**, the root length ratio of fine root to coarse root **(C)**, coarse root surface area **(D)**, fine root surface area **(E)**, the root surface area ratio of fine root to coarse root **(F)**, and the root volume ratio of fine root to coarse root **(G)** of *D. odorifera*. Different lowercases in each graph indicate significant difference among different competitive models (competition between D. odorifera and various N-fixing niche neighbors) under the same water and N application according to Duncan’s tests (p < 0.05). Different uppercases in each graph indicate significant difference among different water and N fertilization application in the same competitive models according to Duncan’s tests. For abbreviations explanation of treatments (species competition models, water, and N fertilization), data description, and statistics are the same as shown in Figure 1.

Under both the 100% FC and 30% FC conditions, *D. odorifera* plantlets showed a greater root length and surface area of both coarse and fine roots in the interspecific competition (Do-Dr and Do-Sm) than intraspecific competition (Do-Do) model with root system contact (Figures 3A,B,D,E). Compared to the well-watered condition, drought stress decreased both the root length and surface area of coarse roots, but it promoted the root length and surface area of fine roots, the root length ratio of fine root to coarse root, the surface area ratio of fine root to coarse root, and the volume ratio of fine root to coarse root (Figure 3). In addition, these parameters of *D. odorifera* showed a greater increase with interspecific competition (Do-Dr and Do-Sm) than intraspecific competition (Do-Do). N application promoted the coarse root length of *D. odorifera* in the Do-Do and Do-Sm models but decreased the root length and surface area of fine roots and the root length ratio of fine root to coarse root in all competition models under the well-watered condition. Under the 100% FC+N condition, *D. odorifera* plantlets in the Do-Sm model had the largest root length and surface area of coarse roots but the smallest root length ratio and surface area ratio of fine root to coarse root among all competition models in the root system contact model. However, the opposite was true in the Do-Dr model. N application decreased the root length and surface area of fine roots, the root length ratio, the surface area ratio, and the volume ratio of fine root to coarse root of plantlets in all competition models under drought stress. Moreover, under the 30% FC+N condition, *D. odorifera* plantlets from the Do-Sm model had larger root lengths and surface areas of both coarse and fine roots and a greater volume ratio of fine root to coarse root than those from the Do-Dr model; the values of these parameters in these two models were significantly higher than those in the Do-Do model. Water × competition and N × competition interactions significantly affected the surface area of fine roots. Water × N × competition interaction significantly affected the surface area of coarse roots and the surface area ratio of fine root to coarse root.

Similar to the aforementioned root morphological indicators, the root system classification of *D. odorifera* under the root system isolation model (Do/Do, Do/Dr, and Do/Sm) exhibited few differences by competition model (Figure 3). However, compared to the root contact model, *D. odorifera* in intraspecific competition under the root isolation model had larger root length and root surface area of both coarse and fine roots under the 100% FC, 30% FC, 30% FC+N conditions (Figures 3A,B,D,E). Under the 100% FC+N condition, *D. odorifera* plantlets in the Do/Dr model with root system isolation showed larger root length and root surface area of coarse roots but a smaller root length ratio and surface area ratio of fine root to coarse root compared to those in the root system contact model (Do/Dr vs. Do-Dr; Figures 3A,C,D,F).

### Effects on Root Nodules

**Figure 4:**
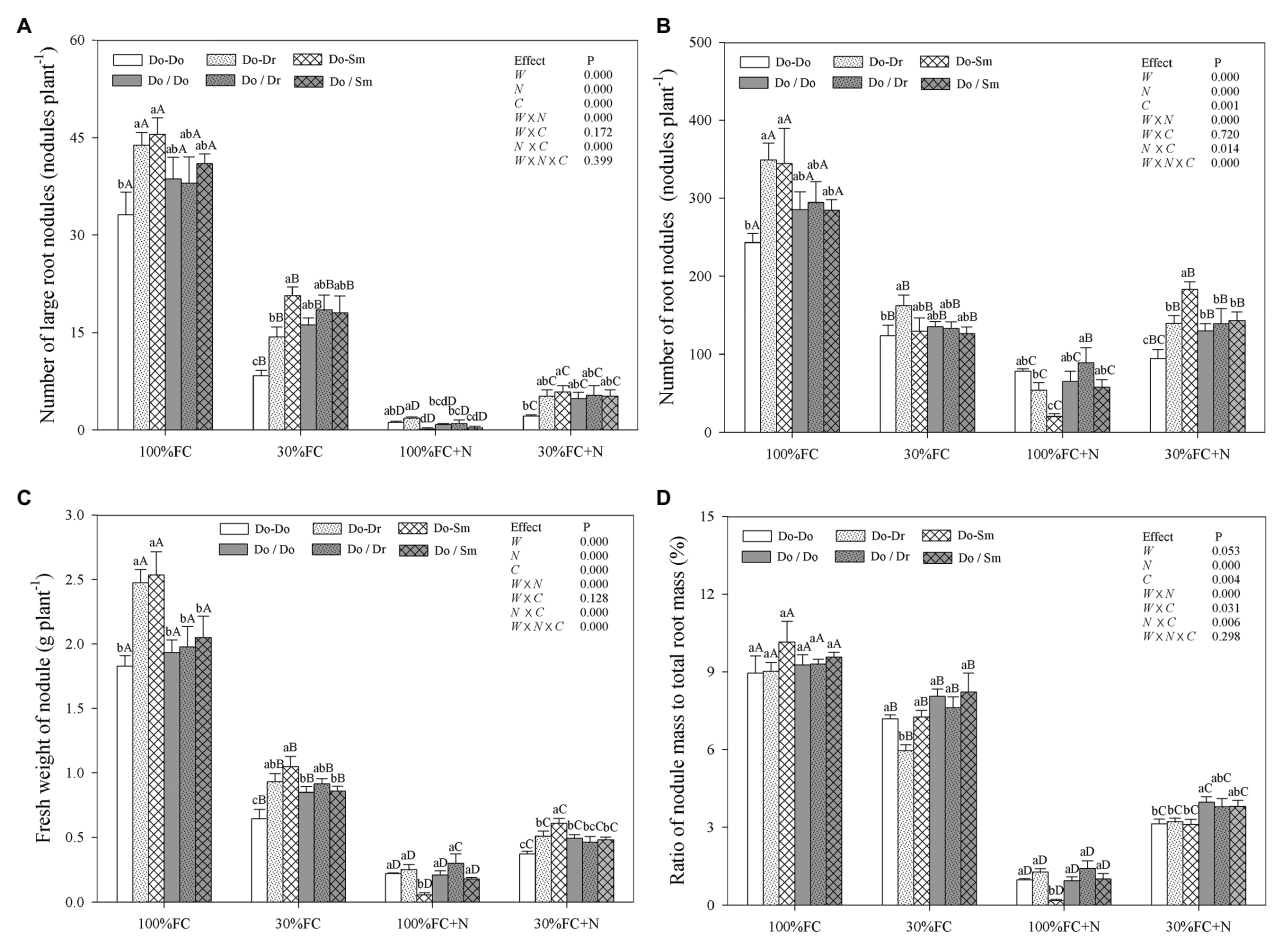
Effects of water, N fertilization levels, and species competition models on large nodules number **(A)**, total nodules number **(B)**, fresh nodules weight **(C)**, and the ratio of nodules mass to root mass **(D)** of *D. odorifera*. Different lowercases in each graph indicate significant difference among different competitive models (competition between D. odorifera and various N-fixing niche neighbors) under the same water and N application according to Duncan’s tests (p < 0.05). Different uppercases in each graph indicate significant difference among different water and N fertilization application in the same competitive models according to Duncan’s tests. For abbreviations explanation of treatments (species competition models, water, and N fertilization), data description, and statistics are the same as shown in Figure 1.

Under the 100% FC condition, *D. odorifera* plantlets in the Do-Do model had the smallest number of large root nodules and total root nodules and the smallest fresh weight of root nodules. However, these parameters showed their highest values among the competition models in plantlets in the Do-Dr and Do-Sm models (Figures 4A–C). Drought stress decreased the number of large root nodules and total root nodules, the fresh weight of root nodules, and the ratio of nodule mass to total root mass (Figure 4). Drought-stressed *D. odorifera* in the Do-Do model showed the smallest number of large root nodules and total root nodules and the smallest fresh weight of root nodules. Although *D. odorifera* in the Do-Sm model had more large root nodules and a higher fresh weight of root nodules than in the Do-Dr model, plantlets in both models with root system contact exhibited a higher number and fresh weight of root nodules than those with root system isolation (Do-Dr vs. Do/Dr, Do-Sm vs. Do/Sm). N application under the 100% FC condition markedly decreased the number of large root nodules and total root nodules, the fresh weight of root nodules, and the ratio of nodule mass to total root mass in all competition models. In addition, *D. odorifera* in the Do-Sm model showed the greatest decreases in these parameters among all competition models. N application under the 30% FC condition markedly decreased the number of large root nodules, the fresh weight of root nodules, and the ratio of nodule mass to total root mass in all competition models (Figures 4A,C,D). Under the 30% FC+N condition, *D. odorifera* in the Do-Do and Do-Sm models had the most and least large root nodules and total root nodules and the highest and lowest fresh weight of root nodules among all competition models, respectively. However, the number of large root nodules and total root nodules, the fresh weight of root nodules, and the ratio of nodule mass to total root mass of *D. odorifera* differed little by treatment with root system isolation. Root nodules were significantly affected by water × N, water × competition, and N × competition interactions. The water × N × competition interaction significantly affected the number and fresh weight of root nodules.

### Correlation Indices of RMP

**Figure 5:**
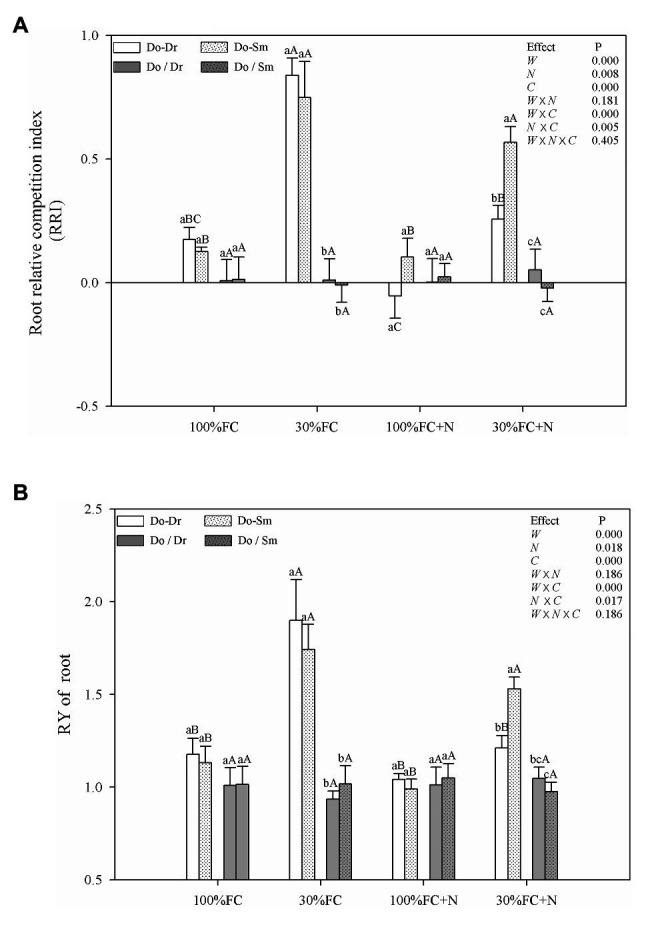
Effects of water, N fertilization levels on RRI **(A)** and RY of root **(B)** in different species competition models. Means ± SEs, *n* = 5. Different lowercases in each graph indicate significant difference among different competitive models (competition between *D. odorifera* and various N-fixing niche neighbors) under the same water and N application according to Duncan’s tests (*p* < 0.05). Different uppercases in each graph indicate significant difference among different water and N fertilization application in the same competitive models according to Duncan’s tests. 100% FC, 100% field capacity; 30% FC, 30% field capacity; 100% FC + N, 100% field capacity and N fertilization; 30% FC + N, 30% field capacity and N fertilization. Do-Dr, Do-Sm indicate *D. odorifera* planting with *D. regia* (white bars) and *S. mahagoni* (white bars with dots) in the root system contact models, respectively; Do/Dr and Do/Sm indicate *D. odorifera* planting with *D. regia* (gray bars) and *S. mahagoni* (gray bars with dots) in the root system isolation models, respectively; W, water factor effect; N, nitrogen factor effect; C, species competition models factor effect; W × N, interaction effect of water and nitrogen factors; W × C, interaction effect of water and species competition models factors; N × C, interaction effect of nitrogen and species competition models factors; W × N × C, interaction effect of water, nitrogen and species competition models factors; Multivariate analysis of variance (Multi-ANOVA) was conducted to evaluate the influence of different factors and their interaction effects.

**Figure 6:**
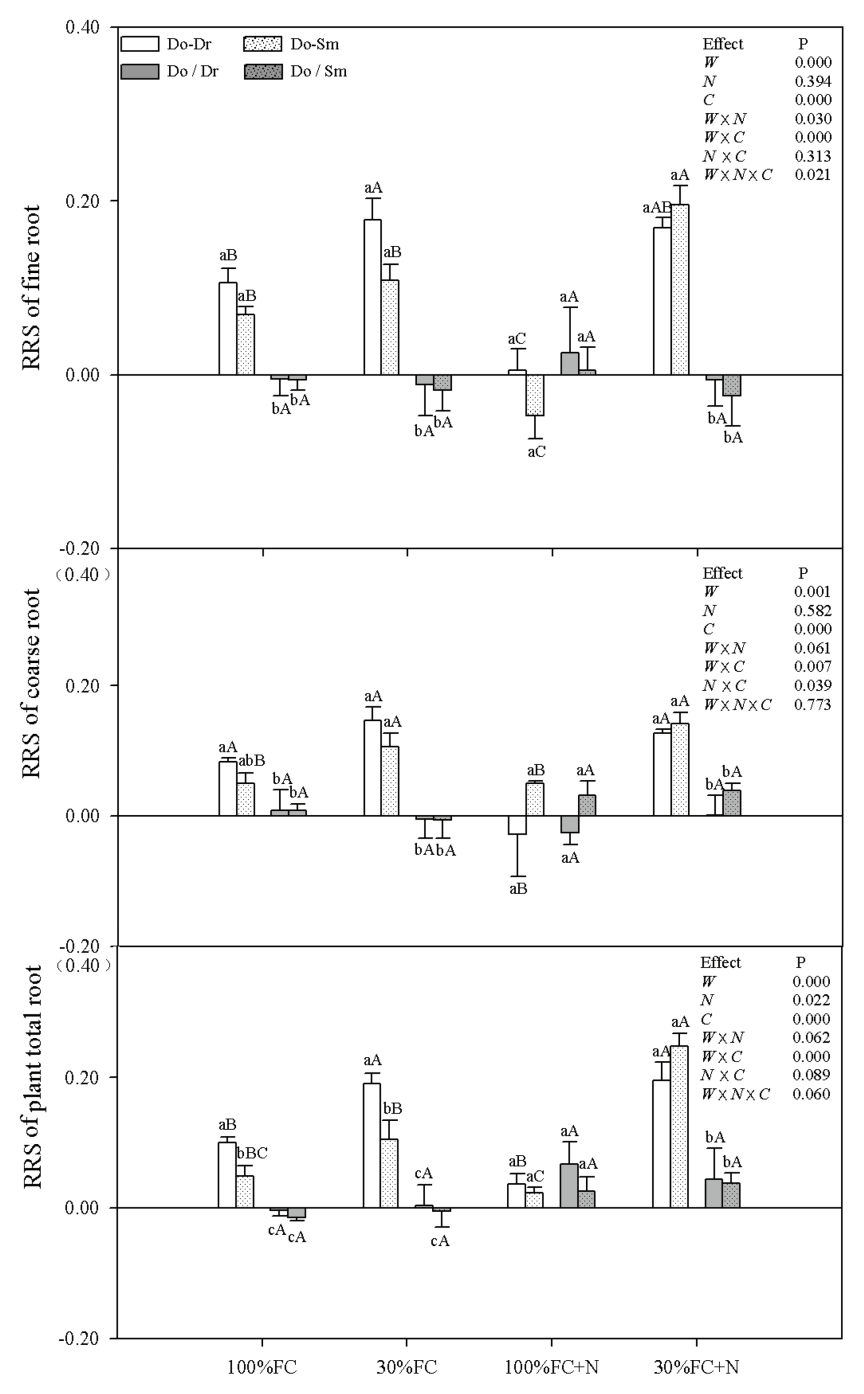
Effects of water, N fertilization levels on RRS of *D. odorifera* in the total root, fine root, and coarse root in different species competition models. Different lowercases in each graph indicate significant difference among different competitive models (competition between D. odorifera and various N-fixing niche neighbors) under the same water and N application according to Duncan’s tests (p < 0.05). Different uppercases in each graph indicate significant difference among different water and N fertilization application in the same competitive models according to Duncan’s tests. For abbreviations explanation of treatments (species competition models, water, and N fertilization), data description, and statistics are the same as shown in Figure 5.

Under the 100% FC and 30% FC conditions, insignificant differences in the RRI and RY of total roots and the RRS of both coarse and fine roots were observed between the Do-Dr and Do-Sm models, whereas the RRI and RRS exhibited positive values, and the RY of total roots was greater than 1.0 (Figures 5, 6). Drought stress promoted the RRI and RY of total roots and the RRS of fine, coarse, and total roots in both the Do-Dr and Do-Sm models with root system contact. N application under the 100% FC condition decreased the RRI of total roots and the RRS of coarse and fine roots of *D. odorifera* in the Do-Dr model, whereas insignificant differences were found in plantlets in the Do-Sm model, except for a decreasing trend in the RRS of fine roots. The RRI of total roots and the RRS of coarse and total roots in the Do-Sm model were positive, whereas *D. odorifera* in the Do-Dr model showed a negative RRI of total roots and RRS of coarse roots under the 100% FC+N condition. N application under the 30% FC condition significantly decreased the RRI and RY of total roots in the Do-Dr model but increased the RRS of fine and total roots in the Do-Sm model (Figures 5, 6). Under the 30% FC+N condition, *D. odorifera* showed a significantly higher RRI and RY of total roots in the Do-Sm model than in the Do-Dr model (RRI, RRS > 0, RY > 1.0). Few differences in the RRI and RY of total roots and the RRS of coarse, fine, and total roots were observed among all competition models in all treatments with root system isolation.

### Plant Growth and the Relative Competitiveness of the Whole *Dalbergia odorifera* Plantlet

**Figure 7:**
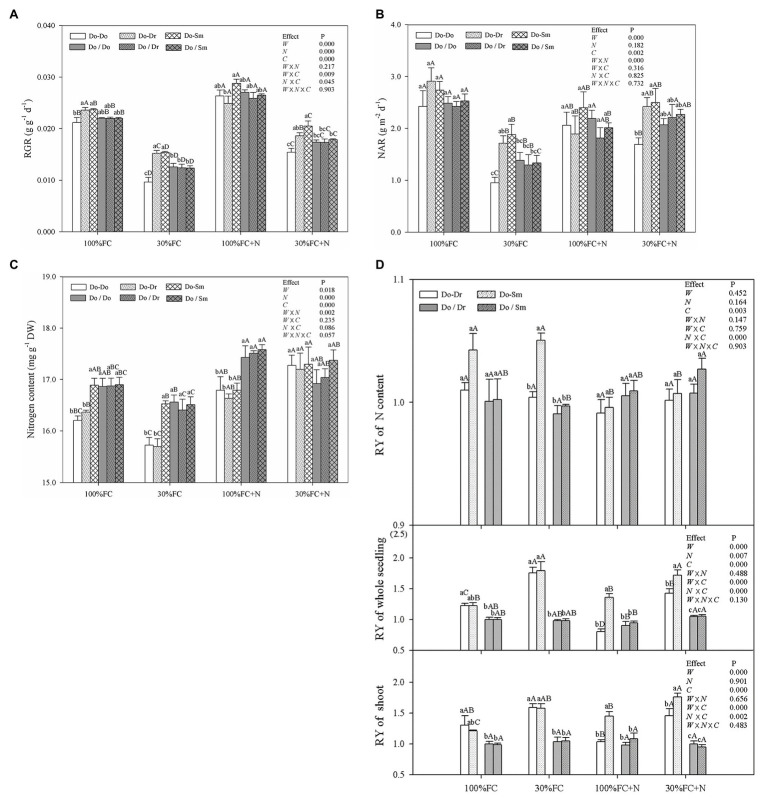
Effects of water, N fertilization levels on RGR **(A)**, NAR **(B)**, leaf N content **(C)** of *D. odorifera*, and RY of the shoot, whole plantlet, and leaf N content **(D)** in different species competition models. Different lowercases in each graph indicate significant difference among different competitive models (competition between D. odorifera and various N-fixing niche neighbors) under the same water and N application according to Duncan’s tests (p < 0.05). Different uppercases in each graph indicate significant difference among different water and N fertilization application in the same competitive models according to Duncan’s tests. For abbreviations explanation of treatments (species competition models, water, and N fertilization), data description, and statistics are the same as shown in Figure 5.

Drought stress significantly decreased the RGR, NAR, and leaf N contents of *D. odorifera* plantlets but increased the RY of shoots and the whole plantlet in all competition models with root system contact (Figure 7). In addition, drought stress caused the greatest decrease in the RGR and NAR of plantlets in the Do-Do model. In contrast, plantlets in the Do-Do model had the smallest RGR, NAR, and leaf N content among all competition models under the 100% and 30% FC conditions; insignificant differences in these parameters were found between the Do-Do and Do-Dr models under the 100% FC condition. Moreover, the RYs of shoots, the whole plantlet, and leaf N content in the Do-Dr and Do-Sm models were greater than 1.0. N application under both water conditions increased the RGR and leaf N content of *D. odorifera* in all competition models but decreased the RYs of shoots and the whole plantlet in the Do-Dr model. *D. odorifera* plantlets in the Do-Dr model had lower values for the RGR, NAR, and RYs of shoots and the whole plantlet than those in the Do-Sm model under the 100% FC+N and 30% FC+N conditions. Moreover, the RYs of shoots and the whole plantlet in the Do-Dr model were less than 1.0 under the 100% FC+N condition. N-applicated *D. odorifera* in the Do-Do and Do-Sm models showed a greater increase in the RGR than those in the Do-Dr model under the 30% FC condition. Moreover, plantlets in the Do-Do model showed the lowest RGR and NAR among all competition models. However, insignificant differences in RGR, NAR, leaf N content, the RY of shoots, the whole plantlet, and N content among the competition models were still observed in all treatments with root system isolation.

### Relationships Between the Relative Competitive Advantage of Roots and the Relative Competitiveness of the Whole Plantlet (Exp1)

**Figure 8:**
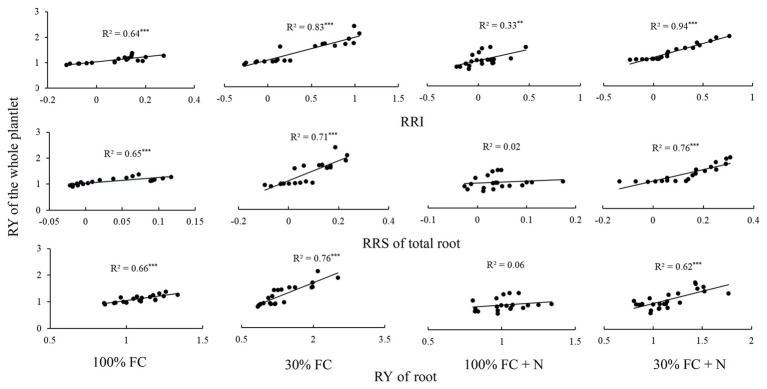
The relationships between the RY of whole plantlet and RRI, RRS, and RY of total root, respectively. One point showed one sample. The solid lines represent the best-fit linear regressions. ^**^p < 0.01; ^***^p < 0.001. 100% FC, 100% field capacity; 30% FC, 30% field capacity; 100% FC+N, 100% field capacity and N fertilization; 30% FC+N, 30% field capacity and N fertilization.

Under the 100% FC condition, the RY of the whole plantlet was significantly positively correlated with the RRI, RY, and RRS of total roots (*p* < 0.05; Figure 8). Moreover, drought stress significantly enhanced these positive correlations. However, N application under the 100% FC condition markedly decreased these correlations. In addition, although a significant correlation was found between the RY of the whole plantlet and the RRI of the roots, an insignificant correlation was observed between the RY of the whole plantlet and the RY and RRS of total roots under the 100% FC+N condition (*p* < 0.05). N application reduced the significant positive correlation between the RY of the whole plantlet and the RY of total roots. The RY of the whole plantlet was positively correlated with the RRI, RY, and RRS of total roots under the 30% FC condition.

## Discussion

### Root Morphology and Plant Growth Respond Differently to Different N-Fixing Niche Neighbors in a Root System Contact Model (Exp1)

Root traits are key indicators of the relative competitiveness of a plant (Goisser et al., 2016), and root plasticity can reflect the utilization of available resources and the adaptability of plants to environmental stress through adjustments to root growth, morphology, and/or physiological activity (Huang and Eissenstat, 2000; Freschet et al., 2013). Thus, a higher RMP would imply a stronger advantage in terms of competition for resources (Huang and Eissenstat, 2000). In addition, roots may respond differently to neighbors (e.g., maximizing root length, root surface area, volume, or diameter) according to the availability of a common resource (Schenk, 2006; Craine et al., 2013). The higher root morphological traits (e.g., root length, root surface area, root diameter, root volume, root tips, root branching number, RRI, and RRS) of *D. odorifera* in interspecific competition compared to intraspecific competition under the 100% FC, 30% FC, and 30% FC+N conditions (Figures 1, 5A, 6) suggest that *D. odorifera* had stronger RMP in the presence of different N-fixing niche neighbors than in the presence of the same one. This finding is consistent with the niche partitioning hypothesis, which argues that plants may reduce root foraging behavior when faced with a niche-similar or equivalent neighboring root because of a similar phenotype or nutrient demand (Macarthur and Levins, 1967; Cheplick and Kane, 2004). A higher RMP increases exploitation efficiency (Huang and Eissenstat, 2000; Richards et al., 2010). Therefore, different N-fixing niches *D. regia* and *S. mahagoni* can increase the exploitation efficiency of leguminous *D. odorifera* under drought stress or N deficiency. Under the 100% FC+N condition, *D. odorifera* planted with *S. mahagoni* had the highest root traits (root volume and diameter) among the three competing models (Do-Do, Do-Dr, and Do-Sm), whereas the lowest levels were observed in plantlets planted with *D. regia*. These findings demonstrate that root morphological traits of *D. odorifera* depend on its N-fixing niche neighbors.

Coarse and fine roots play different roles in function. Coarse roots predominantly store excess carbon, and fine roots absorb belowground resources (Guo et al., 2008). Moreover, fine roots are determinants of the acquisition of soil nutrients and water (Ma et al., 2018). In this study, competition with a heterogeneous neighbor markedly improved the root traits (e.g., root length, root surface area, and RRS) of both the coarse and fine roots of *D. odorifera* than the competition with a conspecific neighbor under the 100% FC, 30% FC, and 30% FC+N conditions (Figures 3A,B,D,E). Moreover, the root length ratio and root surface area ratio of fine root to coarse root of *D. odorifera* benefitted slightly from a heterogeneous neighbor under the 30% FC condition (Figures 3C,F). A greater increase in the lateral fine roots (e.g., root length) of *Picea sitchensis* grown in a mixture with *P. sitchensis* compared to pure stands implies an improvement in exploitation efficiency (Richards et al., 2010). Therefore, the findings indicate that a neighbor with a different N-fixing niche induces more carbon storage in leguminous *D. odorifera* by increasing water and nutrient absorption in a harsh environment. In addition, *D. odorifera* planted with *S. mahagoni* had the largest coarse root length and surface area among all competition models under the 100% FC+N condition, whereas *D. odorifera* planted with *D. regia* had the smallest. These findings might highlight the fact that the resource forage ability and carbon storage of *D. odorifera* are enhanced by *S. mahagoni* but inhibited by *D. regia*.

N-fixing rhizobia are essential for the establishment of symbiotic N fixation in Leguminosae and may mediate competition (Bowsher et al., 2017). Previous studies have demonstrated that nodules can increase both root length and contact area with soil and alleviate soil resource limitations of plant growth by enhancing the acquisition of sparse water and nutrients (Richards et al., 2010). Our results show a higher number of nodules and weight of *D. odorifera* in the interspecific competition models compared to the intraspecific competition models under the 100% FC, 30% FC, and 30% FC+N conditions (Figure 4). These results suggest that the leguminous species in the mixture show greater nodulation than those in monoculture; similar results were observed in studies on an alfalfa–maize intercrop (Wang et al., 2019). Differences in root symbiosis and nutrient preferences between different species in the mixture may lead to greater soil nutrient capture and absorption than in a monoculture. Moreover, there is less competition for resources in mixed cultures (Richards et al., 2010). Therefore, the presence of different N-fixing niche neighbors may benefit the growth of nodules, promoting the absorption of water and nutrients in harsh environments. However, a large number of studies have shown that nodules depend on the availability of soil nutrients, and their distribution can be used to measure plant N fixation strategies (N fixation and soil N uptake; Wang et al., 2019). For example, symbiotic N fixation can be converted to soil N absorption with sufficient N application, thereby reducing rhizobium symbionts (Markham and Zekveld, 2007; Wang et al., 2019). The least number of nodules among all competition models was observed in *D. odorifera* planted with *S. mahagoni* under the 100% FC+N condition (Figure 4), which indicates that sufficient N decreases the number of nodules. The presence of the non-N-fixing niche *S. mahagoni* impacted the availability of soil resources due to differences in plant demand for nutrients and water, further aggravating the N sufficiency. Similar to observed modifications in root morphological traits, these findings emphasize that N-fixing nodules of the leguminous *D. odorifera* can be modulated by a different N-fixing niche neighbor.

### Root and Plant Growth Respond Similarly to Different Niche Neighbors in a Root System Isolation Model (Exp2)

Through both aboveground mechanisms (e.g., light quality signals, volatile organic compounds) and belowground mechanisms (nutrient levels, soluble root exudates), plants can detect and identify their neighbors and estimate their impact on the availability of resources (Pierik et al., 2013). However, plants are exposed to multiple stressors and compete for above- and belowground resources simultaneously in nature. Thus, plant functional traits are changed by mechanisms of both aboveground and belowground interaction (Freschet et al., 2013). In the present study, under the 100% FC, 30% FC, and 30% FC+N conditions, *D. odorifera* in interspecific competition had weaker root plasticity (e.g., lower values for root length; surface area of the total, coarse, and fine roots; root diameter; root volume; branching number) with root system isolation than with root system contact, but similar or stronger root plasticity was found for intraspecific competition (equal or higher values for the aforementioned indices). However, with root system isolation, root morphology and growth (RGR, NAR, leaf N content, and RY of shoots, the whole plantlet, and N content) responded similarly to intraspecific and interspecific competition under all conditions, which indicates that the RMP and growth of *D. odorifera* do not respond to interactions with different N-fixing niche neighbors. Our findings suggest that belowground interaction may be the only way to influence root interaction and the growth of *D. odorifera*, as the effects of aboveground interaction are negligible.

### Effects of Drought and N Application on the RMP of *Dalbergia odorifera* in a Root System Contact Model (Exp1)

Many recent studies have indicated that climate change (e.g., drought and N deposition) can alter the competitive environment of target species through the performance of current competitors (Gilman et al., 2010; Calama et al., 2019). In the present study, drought stress promoted the RMP and root competitiveness of *D. odorifera* against different N-fixing niche neighbors (e.g., a greater increase in root length, root tips, and branching number). Similar responses have been found in other studies, such that facilitative effects of a neighboring plant became stronger under stress (Bertness and Ewanchuk, 2002; He et al., 2013). For example, in one study, the competitive effect of neighbors of *Arrhenatherum elatius* was transformed into a facilitative effect when the plants were exposed to drought (Grant et al., 2014). These results are consistent with the stress gradient hypothesis, which states that positive interaction becomes stronger as the severity of the environment increases (Callaway et al., 2002; Ulrich et al., 2018, 2019). Furthermore, studies have confirmed that severe resource limitations can convert negative interactions to positive ones (Maestre and Cortina, 2003; He et al., 2013; Grant et al., 2014). It is interesting that in this study N application under well-watered conditions converted the promoting effect of *D. regia* on *D. odorifera* RMP (RRI > 0) to an inhibitory effect (RRI < 0). Therefore, N application may alter the root interaction between *D. odorifera* and *D. regia* under well-watered conditions. However, N application under drought stress reduced the root foraging performance of leguminous *D. odorifera* (e.g., decreased the root length, root surface area, root tips, and root branching number). Furthermore, greater decreases in these parameters were observed in the Do-Dr model than in the Do-Do and Do-Sm models, which indicates that N application weakens the promoting effect of *D. regia* on RMP under drought stress. Taken together, these results suggest that drought stress and N application might change the RMP of *D. odorifera* exposed to a different N-fixing niche neighbor with root system contact.

### Linking RMP to the Relative Competitiveness of the Whole Plantlet

The RGR and RY are two indicators of the competitive ability of a target species (Ravenek et al., 2016). Plants alter their NAR to improve their adaptability in response to aboveground and belowground competition (Marvel et al., 1992; Simon et al., 2014). *D. odorifera* had a higher RGR and NAR in interspecific competition than in intraspecific competition, and RY values for shoots, the whole plantlet, and leaf N content were greater than 1.0 (Figures 7A,B,D) under the 100% FC, 30% FC, and 30% FC+N conditions. These results suggest that the relative competitiveness of *D. odorifera* benefits from different N-fixing niche neighbors, in particular under conditions of drought, N deficiency, and combined drought and N deficiency.

A higher RRS and RRI can contribute to the effective capturing of water and nutrients, and both can be used to estimate neighbor-induced RMP and root competitive advantage (Li et al., 2013, 2018). *D. odorifera* in interspecific competition exhibited positive values for the RRI of total roots and the RRS of the total, coarse, and fine roots. Moreover, the RY of total roots was greater than 1.0 under the 100% FC, 30% FC, and 30% FC+N conditions (Figures 5, 6). This indicates that a different N-fixing niche neighbor induces stronger RMP compared to the same N-fixing niche neighbor under conditions of drought, N deficiency, and combined drought and N deficiency.

Many studies have documented the close functional coordination between belowground changes and the aboveground response (Belter and Cahill, 2015), and the functional traits of plants are associated with competitive dominance. Thus, traits can be used to predict individual performance and population interaction (Fort et al., 2014; Kraft et al., 2015). Root traits have a greater impact on plant performance than leaf traits and are the most important predictors of the population dynamics (Schroeder-Georgi et al., 2016). For instance, root traits can reflect a plant’s competitive advantage (Li et al., 2018; Semchenko et al., 2018). In our study, we discovered a strong correlation between the RMP and the relative competitiveness of the whole *D. odorifera* plantlet. The fact that *D. odorifera* showed stronger relative competitiveness against a different N-fixing niche neighbor than the same N-fixing niche neighbor under the 100% FC, 30% FC, and 30% FC+N conditions is consistent with the trend toward a higher RMP. In addition, the RY of the whole plantlet was well correlated with the RRI, RRS, and RY of total roots under the 100% FC, 30% FC, and 30% FC+N conditions (Figure 8). Such correlations imply that RMP to different N-fixing niche neighbors might be useful for predicting the relative competitiveness of the whole plantlet in an environment of severe stress. This finding is consistent with a previous study, in which a significant correlation was found between root traits and species performance (Schroeder-Georgi et al., 2016). The finding can be explained by the stronger RMP of *D. odorifera* that resulted from having a complementary N-fixing niche neighbor, which aided in the capture of a large amount of available resources (Lambers et al., 2017; Erktan et al., 2018) and ultimately enhanced the relative competitiveness of the whole plantlet (Li et al., 2018; Semchenko et al., 2018).

## Conclusion

The RMP of leguminous woody *D. odorifera* is promoted by a different N-fixing niche neighbor under conditions of drought, N deficiency, and combined drought and N deficiency with root system contact. Drought stress improves the RMP of *D. odorifera* exposed to a different N-fixing niche neighbor. N application converts the promoting effect of *D. regia* on RMP to an inhibitory effect under well-watered conditions. Moreover, N application under drought stress weakens the promoting effect of *D. regia* on RMP. Belowground interaction with a different N-fixing niche neighbor may be the only way to influence the RMP of *D. odorifera*, as the effects of aboveground interaction are negligible. Furthermore, neighbor-induced RMP might predict the relative competitiveness of the whole *D. odorifera* plantlet under conditions of drought, N deficiency, and combined drought and N deficiency. These findings provide novel insights into neighbor-induced dynamic changes in the root traits of leguminous woody species in forest systems in the context of climate change.

## Data Availability Statement

The original contributions presented in the study are included in the article/supplementary material, further inquiries can be directed to the corresponding author.

## Author Contributions

L-SX, L-FM, and FY contributed to the present work. L-SX performed the experiment and wrote the draft manuscript. L-FM assisted to carry the experiment in the greenhouse. FY designed the experiment, provided funding, and edited and revised the manuscript. All authors contributed to the article and approved the submitted version.

### Conflict of Interest

The authors declare that the research was conducted in the absence of any commercial or financial relationships that could be construed as a potential conflict of interest.

## Abbreviations

NAR: Net assimilation rate
RGR: Relative growth ratio
RMP: Root morphological plasticity
RRI: Root relative competition index
RRS: Root response ratio
RY: Relative yield
SBD: Specific branching density
SRN: Specific root tip number
SRL: Specific root length
